# Rotational manipulation of a microscopic object inside a microfluidic channel

**DOI:** 10.1063/5.0013309

**Published:** 2020-10-27

**Authors:** Hiroyuki Harada, Makoto Kaneko, Hiroaki Ito

**Affiliations:** 1 Department of Mechanical Engineering, Graduate School of Engineering, Osaka University, Osaka 565-0871, Japan; 2 Division of Mechanical Engineering, Graduate School of Science and Technology, Meijo University, Aichi 468-0073, Japan; 3 Department of Physics, Graduate School of Science, Chiba University, Chiba 263-8522, Japan

## Abstract

Observations and analyses of a microscopic object are essential processes in various fields such as chemical engineering and life science. Microfluidic techniques with various functions and extensions have often been used for such purposes to investigate the mechanical properties of microscopic objects such as biological cells. One of such extensions proposed in this context is a real-time visual feedback manipulation system, which is composed of a high-speed camera and a piezoelectric actuator with a single-line microfluidic channel. Although the on-chip manipulation system enables us to control the 1 degree-of-freedom position of a target object by the real-time pressure control, it has suffered from unintended changes in the object orientation, which is out of control in the previous system. In this study, we propose and demonstrate a novel shear-flow-based mechanism for the control of the orientation of a target object in addition to the position control in a microchannel to overcome the problem of the unintended rotation. We designed a tributary channel using a three-dimensional hydrodynamic simulation with boundary conditions appropriate for the particle manipulation to apply shear stress to the target particle placed at the junction and succeeded in rotating the particle at an angular velocity of 0.2 rad/s even under the position control in the experiment. The proposed mechanism would be applied to feedback controls of a target object in a microchannel to be in a desired orientation and at a desired position, which could be a universally useful function for various microfluidic platforms.

## INTRODUCTION

Manipulation of a target object in a microscopic scale is an important technology to achieve object arrangements, incubation of cells and organs, and evaluation of three-dimensional structures and shapes at a single cell level in the fields of chemical engineering and life science. In terms of the position control (trapping) of a microscopic object, optical tweezers,^1–3^ electrophoresis and dielectrophoresis by applying electric fields,^4,5^ flow control,^6–8^ etc., have been used. In addition to the position control, the attitude control of a microscopic object by rotational manipulation is an important topic in particle manipulations. Optical tweezers with asymmetric setups,^3,9–15^ circularly polarized light beams,^16,17^ Laguerre–Gaussian light beams (optical vortex),^18–20^ and electric fields^21–24^ have been applied to rotate the objects with a high refractive index and/or objects with geometrical or optical anisotropy. While the working principle of these methods is the generation of the rotational torque on the target object, generation of rotating flow fields in the surrounding fluids have also been realized using micropipettes^25^ or vibrating boundary walls.^26–32^ In particular, utilizing vortices generated by the acoustic vibration of channel walls or microbubbles is a typical method for rotating a target object in a microchannel.

Owing to recent developments in nanotechnology and microfabrication techniques, microfluidic channels have been widely used for high-throughput assays such as the sorting of microparticles.^33,34^ Another important scope in microfluidics is a high-throughput evaluation of a single-cell mechanics with statistical reliability,^35^ where the cellular deformability is measured typically by observing the deformation during the passage through a narrow constriction.^36–40^ The position control and orientation control with high precision in a microchannel enable the application of force and/or displacement with the precisely controlled magnitude and direction to a target microobject. With the aid of the combination of the position control and the orientation control, repetitive loadings^47^ from desired directions at a narrow constriction can be realized as an example of promising potential applications. In addition to such uniaxially squeezed deformation, applying desired wall shear stress tangential to the object surface can be also realized, enabling to measure the mechanical responses different from that to the squeezing stress normal to the object surface. Furthermore, the combination of the present orientation control with other manipulation techniques such as high-frequency cell deformation under the electric field will be an interesting application, where the cell experiences both the normal and tangential stresses simultaneously. For the application to these evaluations, manipulation techniques within a microchannel are worth developing. For this purpose, active control of flow or target objects in a microchannel has been utilized to extend the limitation of the conventional microfluidics. It has been also reported that the 1 degree-of-freedom (DoF) position of a single cell in a single-line microchannel can be controlled by a visual feedback system.^41^ This system is driven with a feedback loop between a piezoelectric actuator and a high-speed camera, and it has been applied to the measurements of the cellular mechanical properties.^42,43^ In these measurements, the target cell is held at a desired position for a specified time, and its shape change is observed after specified-time deformation in a narrow constriction, which has revealed the cellular deformability depending on the deformation period or frequency.^43–47^ While this system enables the 1 DoF position control, the attitude of a target object is out of control, resulting in unintended rotation of the target object by fluctuation or wall shear stress. Since the mechanical properties are obtained only through image processing of the object shape in this system,^42–47^ changes in the orientation of the target object can cause the evaluation error. Therefore, the attitude control of a target object should be implemented independently of the previous 1 DoF position control for more precise and elaborate microfluidic assays. Although increasing the DoF of the manipulation allows us to access a new aspect of the mechanical properties of a microscopic object, a minute closed space in a microchannel has constrained available mechanisms for the manipulation and reduced its DoF. In this paper, we report a novel 2 DoF manipulation mechanism that can additionally control the orientation of a target object under the 1 DoF position control in a microchannel.

## METHOD

### Device mechanism

**FIG. 1.**
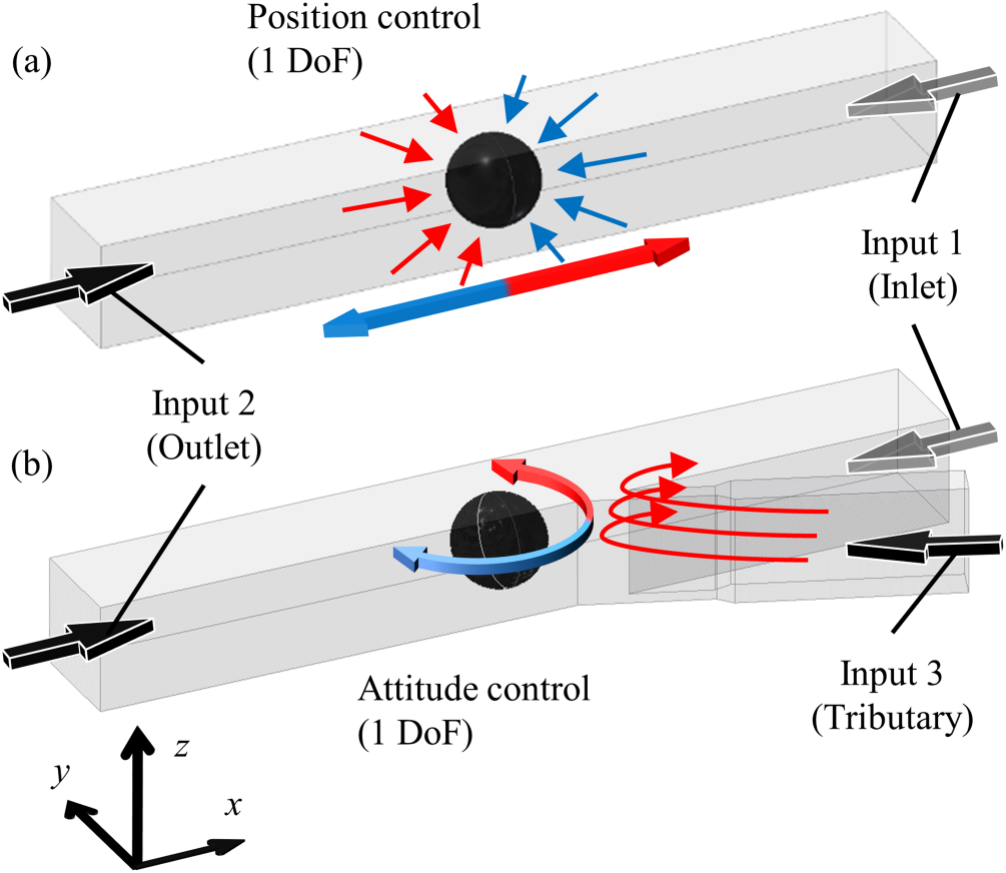
(a) Translational motion of a target object by the pressure input 2 (outlet). (b) Rotational motion of a target object at a fixed position by the inputs 2 and 3 (tributary).

The previous position control system in a microchannel has enabled the 1 DoF control of a target object in a single-line channel.^42–47^ The target object comes into the microchannel from one side [input 1 at the inlet in Fig. 1(a)], and a piezoelectric actuator connected to the other side of the linear channel [input 2 at the outlet in Fig. 1(a)] controls the object position. The actuator performs the real-time and automatic feedback pressure control to adjust the pressure balance between the inlet and the outlet based on the current positions of a target object. The time series of the position is obtained by the real-time analysis of photographs taken by a high-speed camera. For further details of the 1 DoF manipulation, see Refs. 42–47. In the present work, we introduced a new pressure input from a narrow branch channel [input 3 in Fig. 1(b)], called a tributary channel, in addition to the previous pressure inputs in the main channel. The tributary channel is connected to the main channel at an obtuse angle. In general, translational and rotational motions of a microscopic object in a microchannel are driven by the normal and tangential components of a stress tensor at the surface of the target object, respectively. Under the present setup with these pressure inputs, the normal and tangential stresses acting on the target object in a microchannel are mainly originating from the flow in the main channel by the pressure input 2 and the shear flow from the branch channel by the pressure input 3, respectively [Fig. 1(b)]. In particular, the tangential stress only comes from the tilted flow by the input 3. Thus, the system with the additional pressure input can control the orientation of a target object by the input 3, while it is held at a specified position by the position control by the input 2. This mechanism has two remarkable advantages. First, it utilizes only fluid in a microfluidic channel and does not need to introduce other components such as laser beams, electrodes, etc.; the present device only requires the standard soft lithography process in fabrication and one more pressure source. Second, it is applicable to various types of target objects with any material properties (transparency, refractive index, etc.) and/or with anisotropic shapes. One of the limitations of the present system is that the rotational manipulation is limited in a single-line microchannel, where the DoF of translational and rotational motions is reduced by the geometric constraints. In addition to this, uncontrollable small motions along the *y* axis can affect the rotational velocity. Another limitation is that the variety of rotational motions is only around the axis normal to the observed 2D plane.

### Comparison with other mechanisms

We summarize the advantages and disadvantages as well as the characteristics of the target microobject in the present and the typical previous rotational manipulation techniques in Table I. The present method does not require any specific material, shape, and size of the target microobjects since the principle of the hydrodynamic actuation is applicable to any types of microobjects except for that with a high aspect ratio. On the other hand, the rotational manipulation by the optical tweezers requires appropriate optical properties such as dielectricity or birefringence for the target object. It has been applied to various objects with isotropic (sphere^10^ with an anisotropic optical setup) or anisotropic shapes (ellipsoid,^12^ stomatocyte,^13^ fiber,^10^ and chiral objects^14,15^), but it has a limitation in the typical object size up to 10 *μ*m to suppress damages and heating by a high power laser beam (>1 W). Similar to the case of the optical tweezers, the rotation by the external electric fields^21–24^ requires dielectricity of the target object. For the method with vibrating boundaries,^26–32^ a wide range of microobjects are the target of the method because it also utilizes the hydrodynamic force to rotate the particle. However, most of the stable flow and high throughput evaluation in a microchannel is very sensitive to the vibration of the microchannel itself. All their own advantages and disadvantages such as required object properties, the number of axes of rotation, and specially required devices are listed in Table I.

**TABLE I.** Characteristics of various rotational manipulation techniques.

Method	Target microobject (required properties)			Radial velocity (rad/s)	Advantages	Disadvantages
	Material	Shape	Size (*μ*m)			
Present method	Any	Almost axisymmetric shape	1–100	∼0.1	Independent of material properties, Applicable to larger (>10 *μ*m) objects	Inapplicable to high-aspect-ratio objects (rod, wire), 2D in-plain rotation, Require other position control technique
Optical tweezers (tilted Gaussian beam,^9–15^ circularly-polarized beam,^16,17^ Laguerre–Gaussian beam^18–20^)	Dielectricity, Birefringence	Sphere (require anisotropic setup),^10^ ellipsoid,^12^ stomatocyte,^13^ fiber,^10^ chiral,^14^ propeller^15^	1–10	∼0.1	Useful for both trapping and rotating	Heating problem, Limited to the small objects (<10 *μ*m), Require specific material properties (dielectricity, birefringence, etc.) and appropriate anisotropy (ellipsoid, rod, etc.), Expensive setup
Electric field^21–24^	Dielectricity	Any	1–100	∼0.1	3D rotation, Applicable to larger (>10 *μ*m) objects	Require specific material properties (dielectricity), Require other position control technique, Electrostatic interaction
Vibrating boundary^26–32^	Any	Sphere,^28^ Shape with a high aspect ratio (e.g., *C. elegans*)^27^	10–100	∼10–100	3D rotation, applicable to larger (>10 *μ*m) objects, multiple objects,^27^ and flowing objects^27^	Require transducers or oscillators, Limited to large objects (>10 *μ*m)

### Simulation of a rotational motion

First, we designed the microchannel in detail to determine the effective pressure input from the tributary channel for the rotational manipulation. A three-dimensional model to reproduce the flow around the junction of the main and tributary microchannels was created, and the flow field in the microchannel was analyzed by a finite element method (FEM) in COMSOL Multiphysics (COMSOL, Inc., Burlington, MA, USA). Assuming incompressible fluid and laminar flow for the microfluidic flow, the governing equations of the fluid in a steady state are described as

∇⋅v=0, (1)

ρ(v⋅∇)v=−∇p+μΔv, (2)

where ρ is the fluid density, v is the fluid velocity, *p* is the pressure, and μ is the viscosity coefficient of the fluid. The origin of the Cartesian coordinates is set at the center of the spherical particle. Here, we set the conditions for the rotational manipulation of a spherical particle, namely, the conditions for the steady state in which a spherical particle rotates around the *z* axis at a constant angular velocity with no translational motion along the *x* axis. The condition of no translational motion is

$$\int_{S}(\sigma_{xx}+\sigma_{yx}+\sigma_{zx})dS=0,$$ (3)

where $\int_{S}\cdot \;dS$ represents the surface integral over the surface of a particle.$\;\sigma_{ik}$ for i,k=x,y,z is the stress tensor component defined by

$$\sigma =\left(\begin{array}{ccc} \sigma_{xx} & \sigma_{xy} & \sigma_{xz}\\ \sigma_{yx} & \sigma_{yy} & \sigma_{yz}\\ \sigma_{zx} & \sigma_{zy} & \sigma_{zz} \end{array}\right)=\tau -p\left(\begin{array}{ccc} 1 & 0 & 0\\ 0 & 1 & 0\\ 0 & 0 & 1 \end{array}\right),$$ (4)

$$\tau =\mu \left(\begin{array}{ccc} 2\frac{\partial v_{x}}{\partial x} & \frac{\partial v_{x}}{\partial y}+\frac{\partial v_{y}}{\partial x} & \frac{\partial v_{x}}{\partial z}+\frac{\partial v_{z}}{\partial x}\\ \frac{\partial v_{y}}{\partial x}+\frac{\partial v_{x}}{\partial y} & 2\frac{\partial v_{y}}{\partial y} & \frac{\partial v_{y}}{\partial z}+\frac{\partial v_{z}}{\partial y}\\ \frac{\partial v_{z}}{\partial x}+\frac{\partial v_{x}}{\partial z} & \frac{\partial v_{z}}{\partial y}+\frac{\partial v_{y}}{\partial z} & 2\frac{\partial v_{z}}{\partial z} \end{array}\right).$$ (5)

The condition of steady rotation is

$$\int_{S}\left(\tau^{T}n\right)\cdot tdS=0,$$ (6)

where n and t are the unit normal vector to the particle surface and the unit tangential vector to the particle surface in the direction of counterclockwise (CCW) around the *z* axis, respectively. We set the boundary condition for the velocity field v on the particle surface so that v satisfies these conditions. From v=r×ω, where r is the positional vector of the particle surface measured from the origin, we determine the constant angular velocity ω around the *z* axis.

**FIG. 2.**
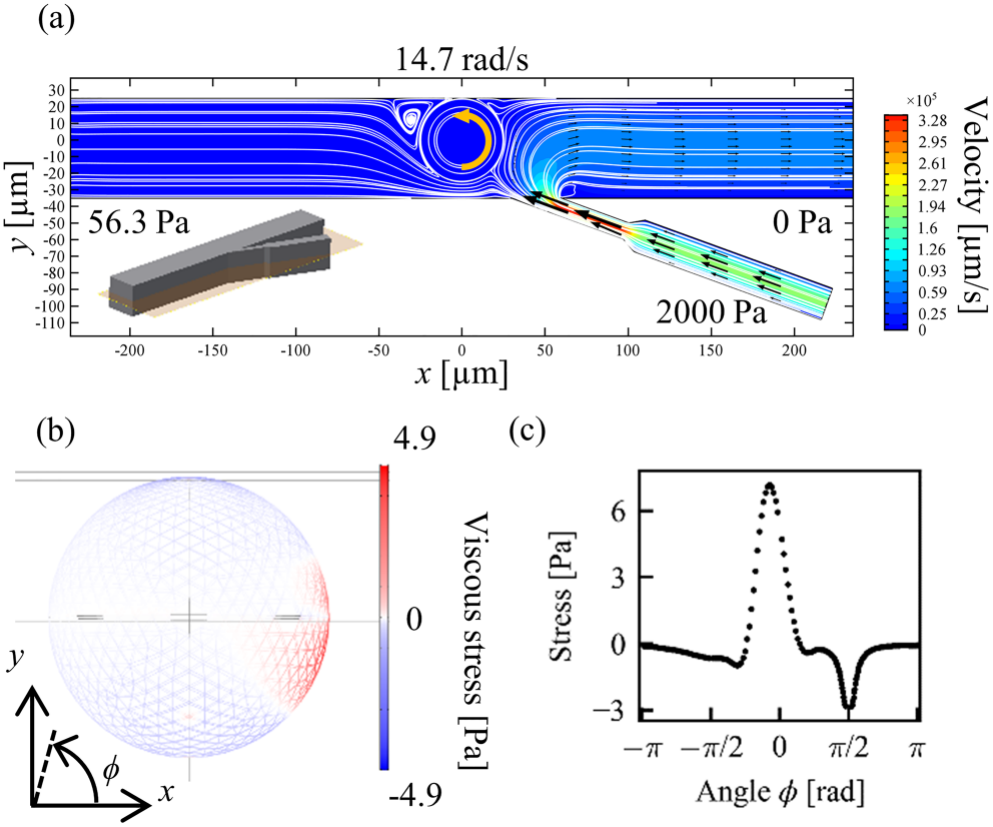
Example of flow simulation with a 3D model of a microfluidic channel. (a) Flow velocity in the *xy* plane. The origin of the Cartesian coordinates is set at the center of the spherical particle. (b) Viscous stress on spherical surface. (c) Viscous stress along the particle equator.

**FIG. 3.**
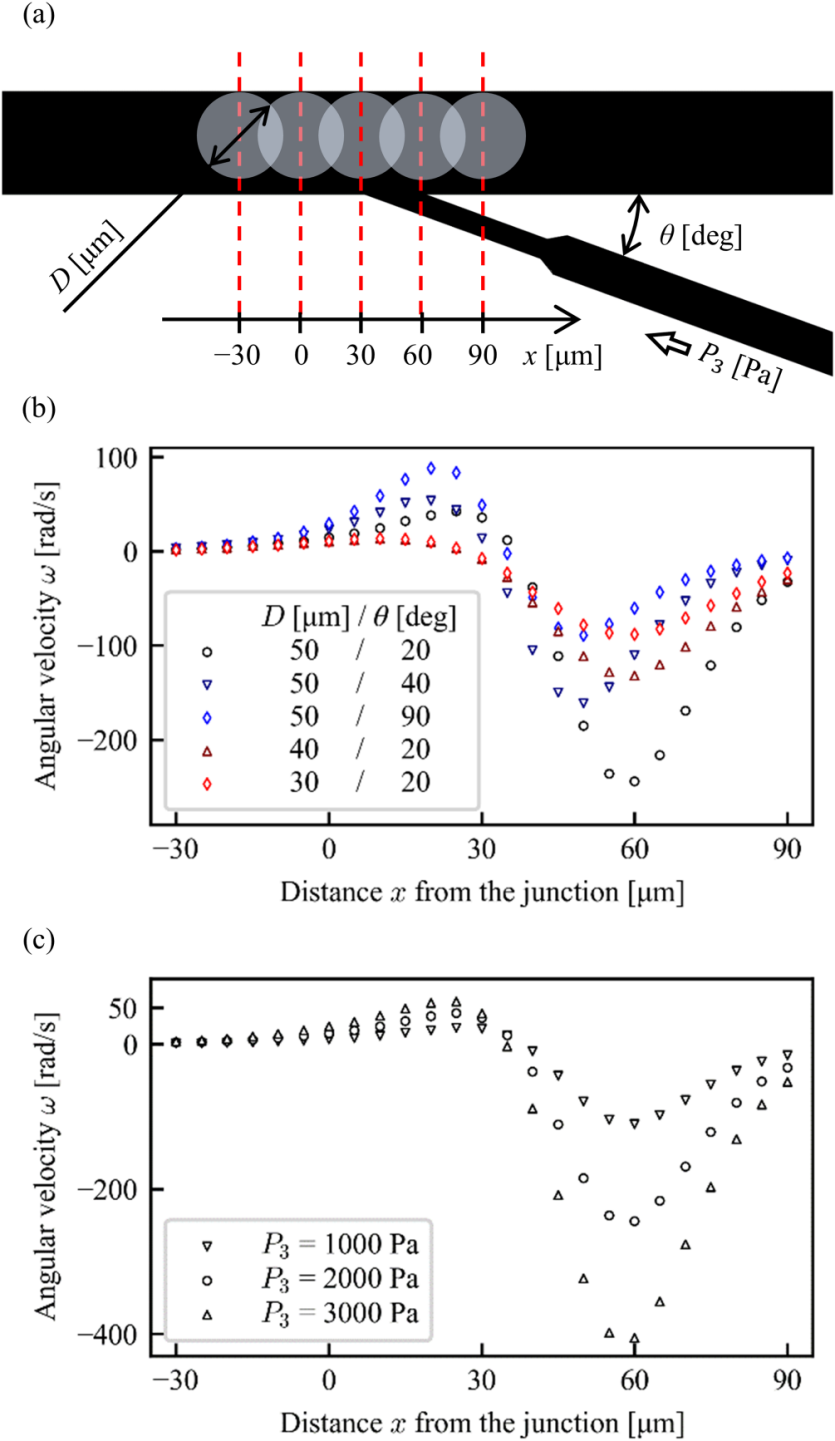
(a) Definitions of the parameters: distance *x* from the tributary junction, angle *θ* of the tributary channel, diameter *D* of the particle, and inlet pressure $P_{3}$ from the tributary channel. (b) Angular velocity *ω* for various *x*, *θ* and *D*. (c) Angular velocity *ω* for various *x* and $P_{3}$.

As the fluid flows into the main channel from the tributary channel, the particle should be pushed toward the channel wall as shown in Fig. 2(a). In general, when a suspended particle moves near a solid surface, fluid always fills between the particle and the solid surface due to the non-slip boundary condition at the both surfaces. In our simulation, the particle–wall distance is set as δ*d* = 0.1 *μ*m, which is the minimum mesh size in a practical simulation as well as the minimum resolution in optical microscopy. More detailed effects of the particle–wall distance will be discussed later. In the simulation, the pressure value from the input 2 is adjusted as a parameter to satisfy the above conditions, while the pressure inputs from the input 1 and the input 3 are fixed at appropriate values. In this setup, the constant input 3, which produces the inclined flow input from the tributary channel, yields shear stress to rotate the target particle. The adjustment of the input 2 against the constant inputs 1 and 3 corresponds to the position control of a rotating target object at a target position. Figure 2 shows an example of a rotating particle at a constant angular velocity ω=14.7rad/s in the direction of CCW at a fixed position in the condition that the relative pressure values of the input 2 and input 3 against the input 1 are 56.3 Pa and 2000 Pa, respectively. Figure 2(a) shows the velocity field and streamlines as a color map and white lines, respectively. Figure 2(b) shows the stress distribution on the spherical surface which indicates the viscous stress in the directions of CCW (red) and clockwise (CW, blue). The red region at around ϕ=0 corresponds to the shear stress by the inclined flow from the tributary channel in the CCW direction, while the other blue region corresponds to the viscous drag in the opposite direction. Note that the surface integral of these stresses is canceled in the condition of steady rotation. Figure 2(c) shows the angular dependence of the stress calculated along the particle equator. The relatively strong viscous stress in the CCW direction at around ϕ=0 and that in the CW direction at around ϕ=π/2 come from the inclined flow and the wall shear stress, respectively. Similar calculations are performed for the different fixed positions of a particle around the junction as shown in Fig. 3(a). The position-dependent angular velocity in each steady state is shown in Figs. 3(b) and 3(c). This result indicates that not only the magnitude of the angular velocity but also the direction of rotation are determined depending on the distance from the junction. If the particle approaches the junction, the magnitude of the angular velocity becomes higher. The purpose of this study is to realize the stable control of the particle orientation. To avoid the unstable motion of a particle with too high angular velocity, the condition of the particle rotation at a constant angular velocity of ω=14.7rad/s at *x* = 0 *μ*m, D = 50 *μ*m, θ = 20°, P3 = 2000 Pa in Fig. 3(a) is adopted in this study.

## EXPERIMENTAL

**FIG. 4.**
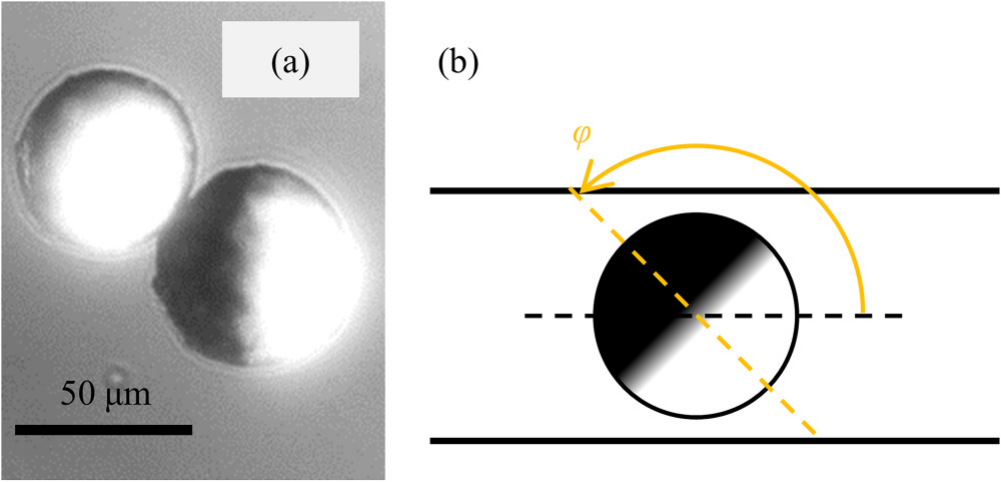
(a) Janus particles. (b) The definition of the orientation angle *φ* of a Janus particle in a microchannel.

**FIG. 5.**
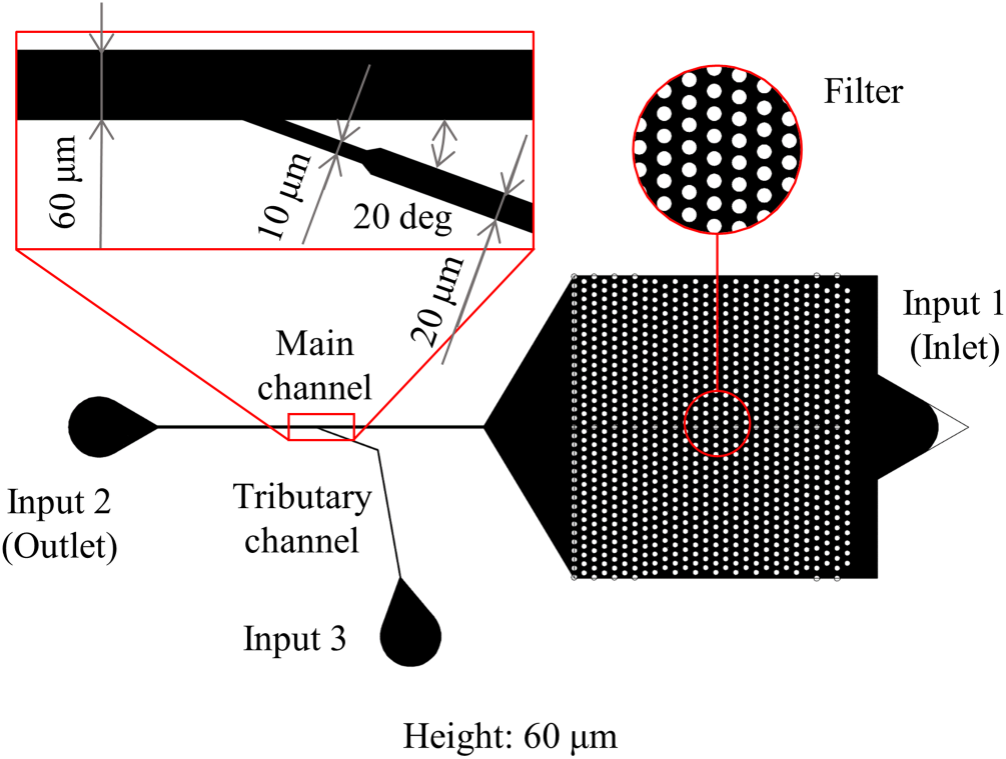
Design of a microchannel for the 2 DoF control system.

To visualize the orientation of a target object, we used a Janus particle (HCMS-BLK-WHT-45-53, Cospheric) with the diameter of 45–53 *μ*m and a distinct color in each hemisphere. Using both reflected-light and transmitted-light observations on a microscope simultaneously, we can observe both the position and orientation of the particle as shown in Fig. 4(a). To determine the attitude of the particle, the orientation angle *φ* in the experiment is set as shown in Fig. 4(b) in a similar manner with that in the simulation. Figure 5 shows the whole design of a microchannel, which includes physical filters to remove debris before the particles enter around the junctional part.

**FIG. 6.**
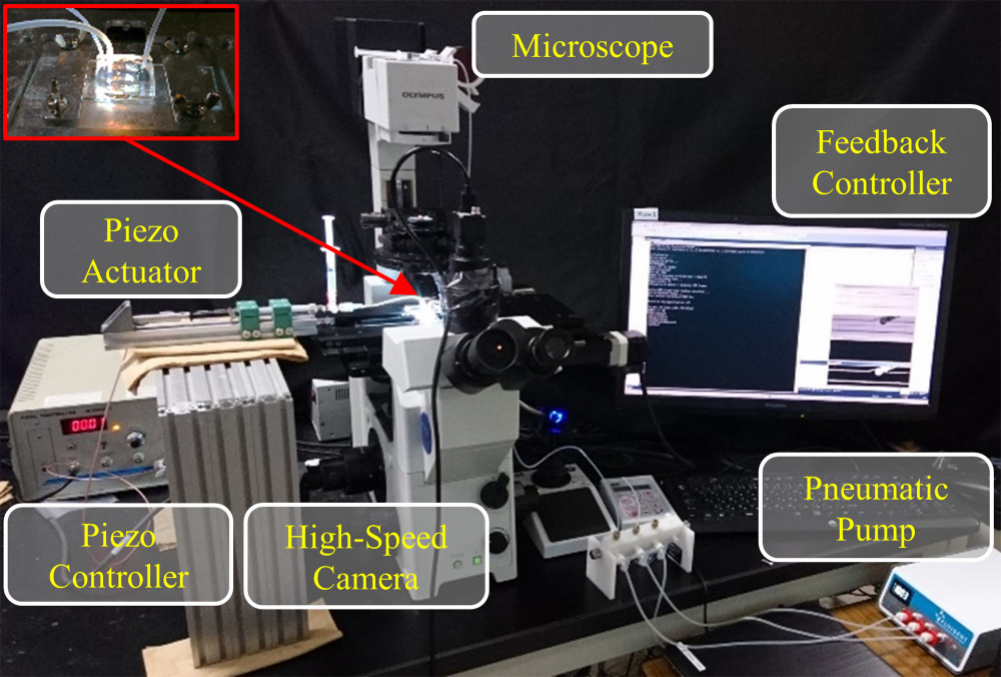
Experimental setup.

We performed the experiment of the rotational manipulation to check the feasibility of the proposed strategy. Figure 6 shows the experimental system. The microchannel is fabricated by casting polydimethylsiloxane (PDMS) onto the mould of an SU-8 photoresist prepared by photolithography. A piezoelectric actuator (PSt 150/5/40VS10, PIEZOMECHANIK) and a pneumatic pump (MFCS™-EZ, Fluigent) are used to control the pressures of the inputs 2 and 3, respectively. For the tributary channel, we adopted a pneumatic pump rather than a piezoelectric actuator with much higher time resolution because the typical flow rate to control the orientation of a target object exceeds the limitation in a short stroke of the piezoelectric actuator. Note that a syringe pump can be also used as the pressure source at the tributary channel, as it has such a longer stroke than the piezoelectric actuator. The Janus particles are introduced from the inlet (input 1), and the flowing particles are observed around the junction. By the real-time analysis of the images taken by a high-speed camera at 1000 Hz, the real-time position and orientation of a target object are obtained. The position control of the target object is achieved by the feedback control of the piezoelectric actuator based on the current position, while the orientation control is achieved by the open loop control of the pneumatic pump. Thus, in the experiment, the pressure input from the pneumatic pump (input 3) is fixed at a constant value to achieve a rotation at a constant angular velocity, and the input from the piezoelectric actuator (input 2) automatically controls the position to suppress translations, as is calculated in the simulation.

To determine the typical scale for the constant pressure value from the pneumatic pump, the pressure loss in the tributary channel before the junction is estimated. The pressure loss Δp of a microchannel with a rectangular cross section is calculated by

$$\Delta p\approx \frac{12\mu QL}{0.79w^{3}h},$$ (7)

where *w* = 20 *μ*m is the channel width, *h* = 60 *μ*m is the channel height, *L* = 3.7 × 10^3^ *μ*m is the channel length, *μ* = 1.0 × 10^−3^ Pa s for pure water at 20 °C, and *Q* is the flow rate.^48^ Considering the simulated values of *Q* = 7.2 *μ*l/min, which is calculated as the surface integral of flow velocity over the cross section of the input 3, the pressure loss is estimated as $\Delta p\approx 1.4\times 10^{4}\;\mathrm{Pa}$. With the fact that the pressure on the input 1 is slightly higher than atmospheric pressure to realize the gentle flow of Janus particles into the microchannel, the typical scale for the pressure value from the pneumatic pump is determined; we fixed it to be 2.0 × 10^4^ Pa in the present experiment.

## RESULTS

**FIG. 7.**
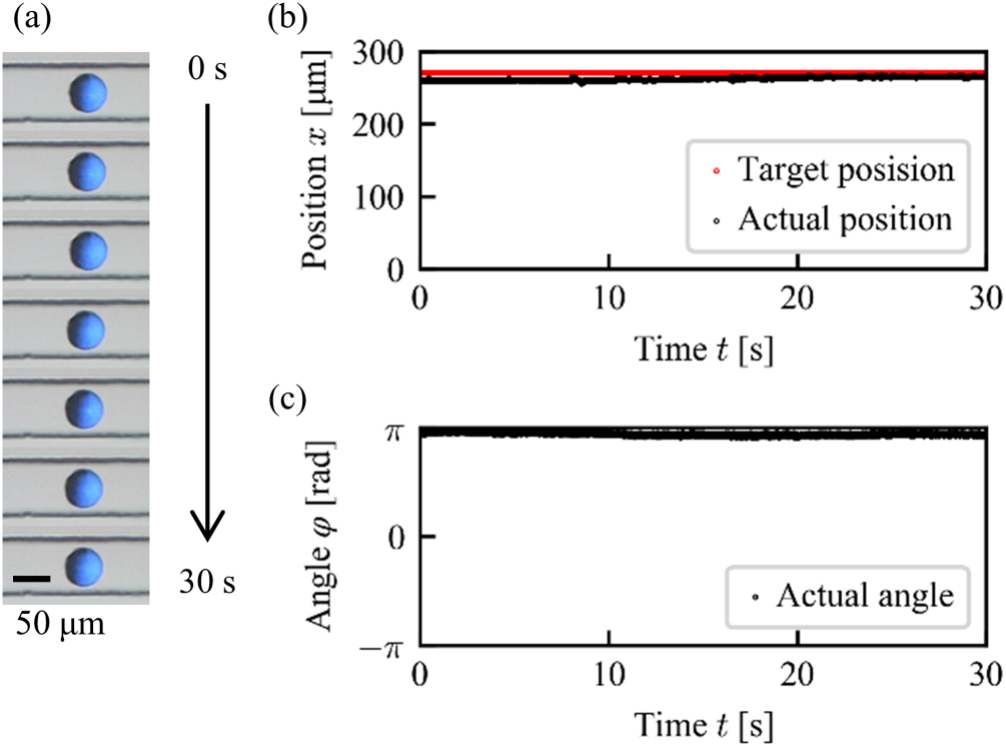
Experimental result for a resting state under 1DoF control. (a) Snapshots. Scale bar: 50 *μ*m. Multimedia view: https://doi.org/10.1063/5.0013309.1. (b) Position. (c) Orientation.
10.1063/5.0013309.1

We first tested whether the position control system works in the main channel even a tributary channel is connected. Figure 7(a) shows the photograph of a Janus particle resting around a fixed target position *x* = 270 *μ*m for 30 s (Multimedia view). Here, we define the particle position *x* in the experiment as the distance measured from the left side of the obtained photographs. The comparison between the target position and the actual position of a particle is shown in Fig. 7(b). This result indicates that the particle position was stably kept at the target position under the feedback pressure control. Figure 7(c) shows the orientation angle of the particle, which also remained almost constant.

**FIG. 8.**
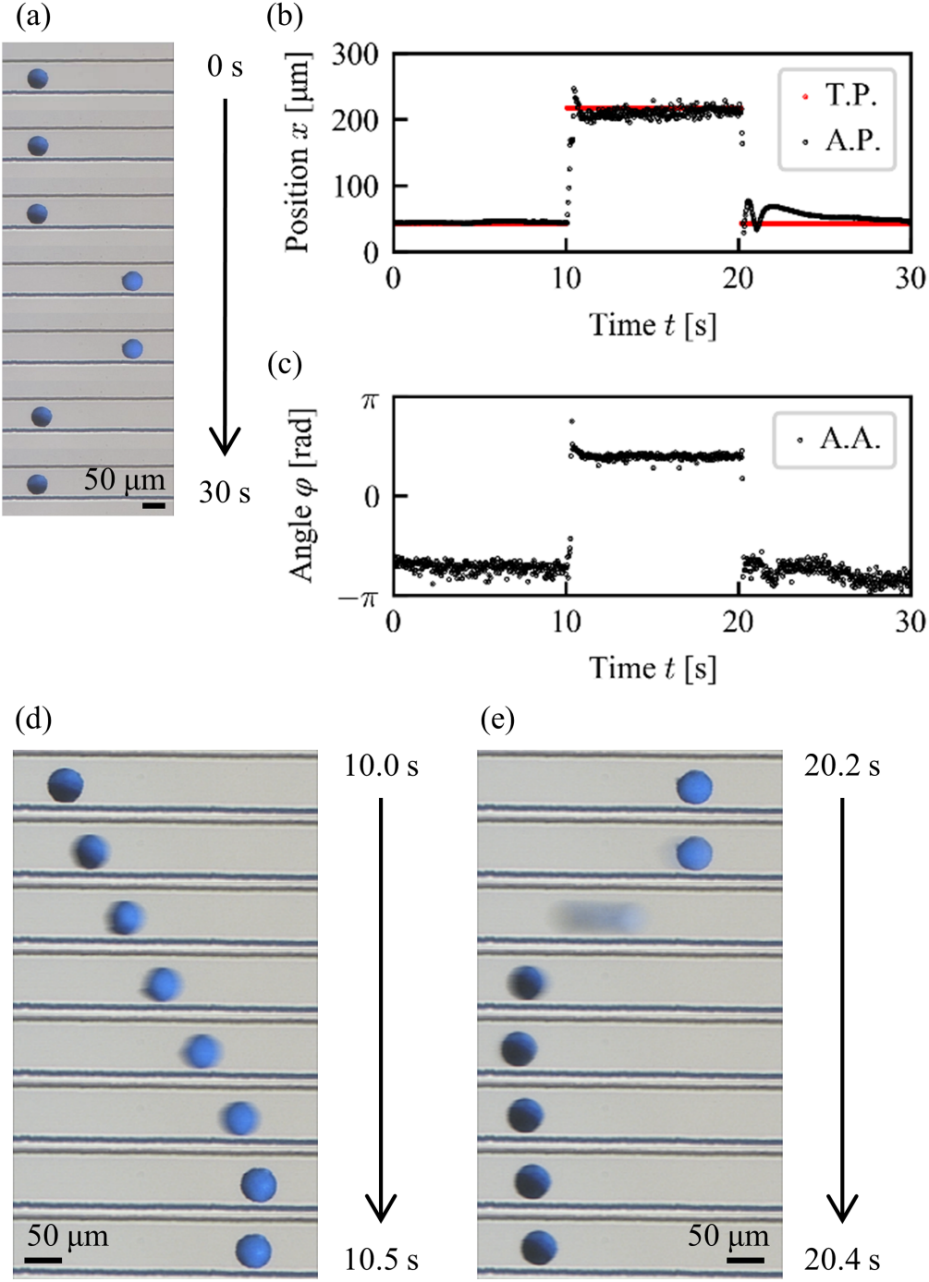
Experimental result for a step motion under 1DoF control. (a) Snapshots. Multimedia view: https://doi.org/10.1063/5.0013309.2. (b) Target position (TP) and actual position (AP). (c) Actual angle (AA). Detailed snapshots of the particle motions toward (d) right and (e) left. Scale bars: 50 *μ*m.
10.1063/5.0013309.2

Next, we measured the change in the particle orientation during the translational motion by the 1 DoF manipulation as shown in Fig. 8. The target position was changed every 10 s as *x* = 45 *μ*m for *t* = 0–10 s, *x* = 220 *μ*m for *t* = 10–20 s, and again *x* = 45 *μ*m for *t* = 20–30 s in a step-like manner. According to the change in the target position, the particle exhibited translational motion in a microchannel as shown in Fig. 8(a) (Multimedia view). The comparison between the target position and actual position of a particle during the motion is shown in Fig. 8(b). Although small overshoots were observed in the actual response, the particle well followed the target position. The corresponding change in the orientation of the particle shown in Fig. 8(c) indicates that the particle orientation was strongly correlated with its position; the particle orientation changed by 3.7 rad in association with the 175 *μ*m translational motion and returned to the original orientation with the inverse translational motion to the original position. A small damped oscillation after the overshoot of the orientation was also observed as in that of the position. It is noted that clear effects of the rotary inertia against this rotational motion were not observed. Figures 8(d) and 8(e) show the detailed snapshots for these forward and backward motions. Due to the strong correlation between the position and the orientation, the particle attitude is uniquely determined at a given particle position. Therefore, it is verified to be impossible to control both the position and orientation of a target object independently by the pressure control system only with the inputs 1 and 2.

**FIG. 9.**
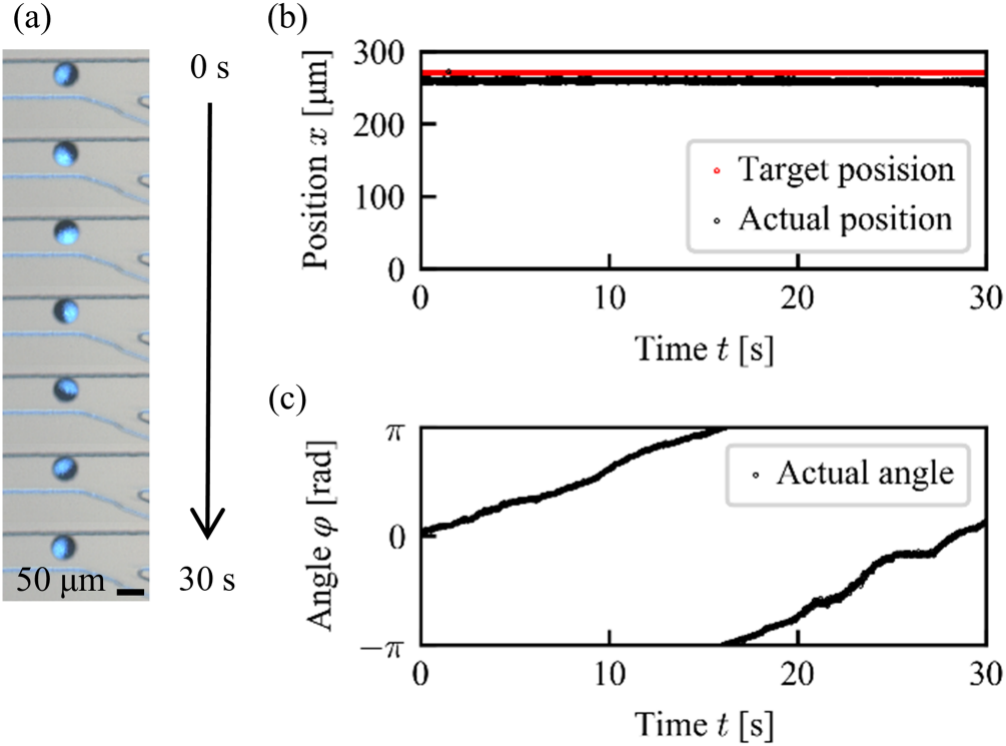
Experimental result for a steady state rotational manipulation under the 2 DoF control system. (a) Snapshots. Scale bar: 50 *μ*m. Multimedia view: https://doi.org/10.1063/5.0013309.3. (b) Position. (c) Orientation.
10.1063/5.0013309.3

Next, we performed the experiment with the 2 DoF control system, in which an additional shear flow is introduced from the tributary channel. The particle was trapped at the junction by the feedback position control as determined in the simulations. Figure 9 shows the results of the 2 DoF control by applying the constant pressure of 2.0 × 10^4^ Pa with a pneumatic pump to the input 3. Figure 9(a) clearly demonstrates that the target particle was rotated by the shear flow from the tributary channel while the position stayed at the junction point (Multimedia view). Figures 9(b) and 9(c) show the changes in the particle position and the orientation during the manipulation, respectively. The particle position was fixed at *x* = 270 *μ*m for 30 s and the orientation changed with the rotational motion at an almost constant angular velocity of ω=0.2rad/s. As the area of the bright part of the Janus particle observed in Fig. 9(a) was also kept almost constant, the observed rotational motion was found to be constrained around the *z* axis. This result reveals that the proposed 2 DoF control system can independently control the orientation of a target object around the axis perpendicular to the channel plane in a microchannel without the change in the position.

## DISCUSSION

**FIG. 10.**
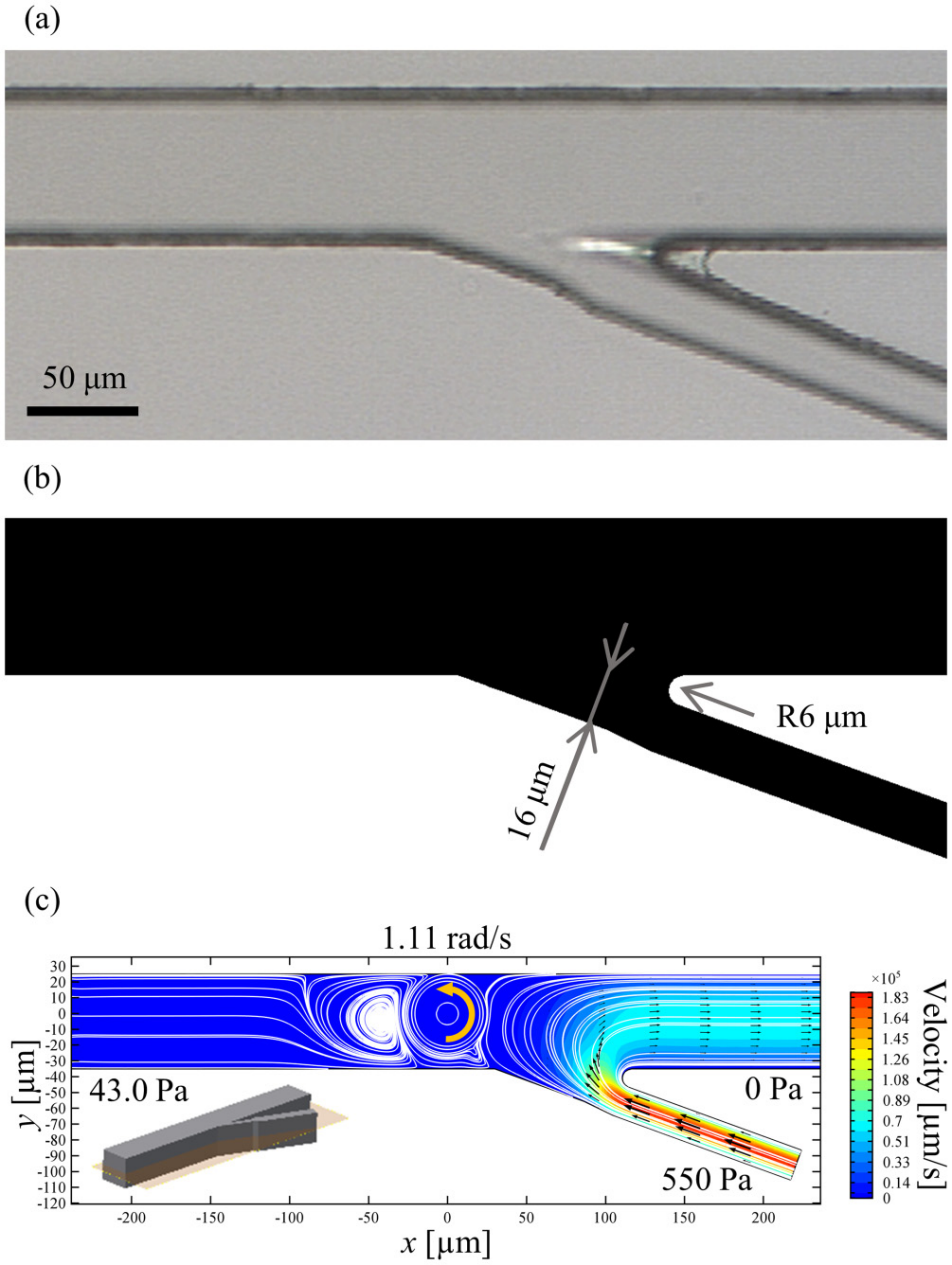
Effect of fabrication accuracy. (a) Magnified photograph of an actual microchannel, where the narrow part is wider than the original design (see Fig. 3). (b) Dimension of a re-designed microchannel. (c) Simulation result.

The orientation of a particle is controlled under the 2 DoF control system in the experiment. However, there is a quantitative difference between the simulation and the experiment in the angular velocity of the particle rotation. Fabrication accuracy of a microchannel can be considered as one of the factors to cause the difference. The actual shape of a microchannel [Fig. 10(a)] became dull compared with its original design (Fig. 3), and the actual width of the narrowest part was about 16 *μ*m, which should have been 10 *μ*m as in the original design. To confirm the effect of the fabrication accuracy, we additionally performed another FEM simulation using an alternative design [Fig. 10(b)] that reflects the fabricated shape of a microchannel under almost the same flow rate *Q* through the input 3 with that in Fig. 4(a). Figure 10(c) shows the result of the simulation under relative pressure differences of the input 2 and the input 3 against the input 1 are 43.0 Pa and 5.5 × 10^2^ Pa, respectively. It indicates that a particle rotates at a constant angular velocity of 1.11 rad/s in the direction of CCW, which is the same order of magnitude with the angular velocity measured in the experiment, with no translational motion.

**FIG. 11.**
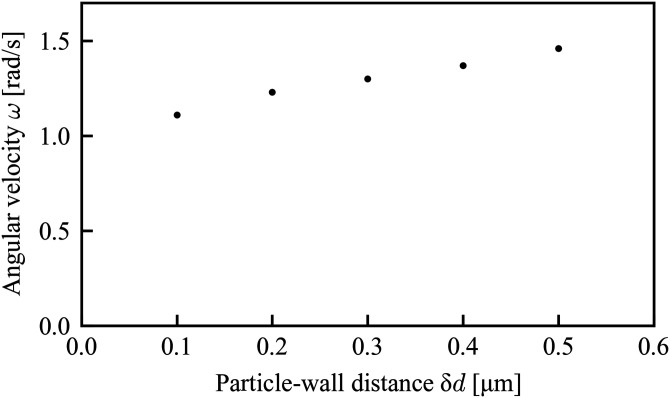
Dependence of the angular velocity on the distance between the particle and the wall of the microchannel.

In addition to the fabrication accuracy, the distance between the sidewall of a microchannel and the particle, as well as the distance between the floor and the particle, which can vary by the sedimentation, can be also considered as other factors for the angular velocity difference. First, we evaluated the effect of the distance between the sidewall and the particle. In general, the closer the particle-wall distance is, the stronger the frictional shear stress yielded on the particle surface is. Thus, the rotation of the particle near the wall tends to be suppressed. Although it is impossible to measure the actual particle-wall distance from the snapshots shown in Fig. 9 because of the limitation in optical resolution, the correlation between the particle-wall distance δ*d* and the angular velocity of particle rotation ω can be found from the simulation results with the re-designed microchannel as shown in Fig. 11. Considering the smooth function and the non-slip condition for the particle-wall surfaces, i.e., *ω*(δ*d* = 0) = 0 rad/s, the angular velocity *ω* = 0.2 rad/s measured in the experiment was obtained in the distance smaller than δ*d* = 0.1 *μ*m. The range estimated here is smaller than the optical resolution, which are consistent with the actual situation.

**FIG. 12.**
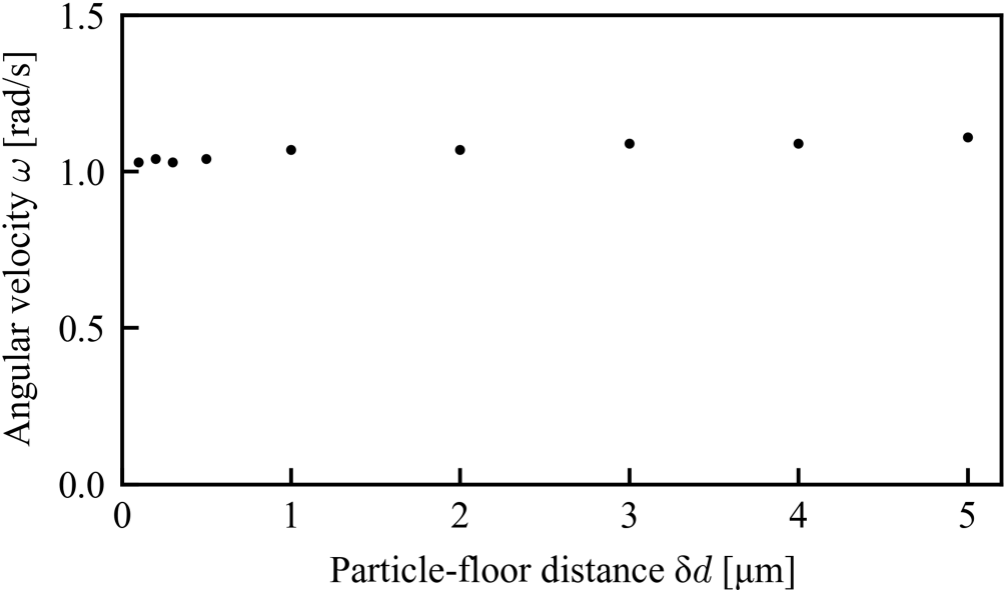
Dependence of the angular velocity on the distance between the particle and the floor of the microchannel.

Next, we evaluated the effect of the distance between the floor and the particle. As we can see in Figs. 8(d) and 8(e), three-dimensional rotation was induced during the translational motion in the experiment, which indicates the existence of the wall shear stress between the floor and the particle due to the sedimentation of the target particle. Focusing on the rotational motion at a fixed position, on the other hand, the rotational motion shown in Fig. 9 seems to be only around the axis normal to the observed 2D plane. Although the actual distance between the floor and the resting particle is not accessible in the experiment, we evaluate the angular velocity under such wall shear stress in the simulation. We considered the spacing between the floor and the particle from 0.1 *μ*m to 5 *μ*m, and obtained the numerical solution. The result is shown in Fig. 12, which exhibits much less dependence of the angular velocity on the spacing compared to that on the particle-wall distance δ*d* shown in Fig. 11. This result indicates that the sedimentation has little observable effect on the rotational manipulation.

In the present work, we focused on the working principle of the rotational manipulation in a microfluidic channel, and real-time control is not yet implemented. As the orientation of the particle was changed with the rotational motion at an almost constant angular velocity in the experiment (see Fig. 9), it will be possible to set a specific angle of the target particle in a real time manner by introducing a visual feedback mechanism between the orientation of a particle and the pressure input from the tributary channel. The resolution of such feedback rotational manipulation can be, in theory, evaluated by the bottleneck of the control period of the pneumatic microfluidic pump (40 ms). Given that the angular velocity of the particle is ∼0.2 rad/s as in the experiment, the angle can be set with the resolution of 0.008 rad (0.46°). For comparison, the angular velocities obtained in the other previous methods are similar (∼0.1 rad/s) for the optical tweezers,^9–20^ electric fields,^21–24^ and vibrating walls^28^ and much faster (∼10–100 rad/s) for the vibrating bubbles^26,31^ (see Table I).

## CONCLUSION

In this study, we proposed a novel microfluidic system for the control of the orientation as well as the position of a target object in a microchannel by improving the previous 1 DoF position control system. We utilized the tributary channel to apply shear stress to the target particle placed at the junction. Based on the design principle developed with the FEM simulation, we experimentally demonstrated the simultaneous controls for both the position and orientation of a Janus particle. The angular velocity in the rotational motion of a particle under the 2 DoF control system was about 0.2 rad/s in the experiment. Although it was two orders of magnitude smaller than that obtained in the simulation, the corrections regarding the practical channel dimension and the particle–wall distance in the simulation well reproduced the experimental measurement. The potential application of the present 2 DoF control system would be a feedback control of a target object to be in a desired orientation and at a desired position in a microchannel, which could be a universally useful function for various topics of microfluidics.

## AUTHORS’ CONTRIBUTIONS

All authors designed the study. H.H. and H.I. performed the experiments and analyzed the data. All authors participated in discussion and manuscript preparation.

## Data Availability

The data that support the findings of this study are available from the corresponding author upon reasonable request.
